# Postoperative acute kidney injury increases short- and long-term death risks in elderly patients (≥ 75 years old) undergoing coronary artery bypass graft surgery

**DOI:** 10.1007/s11255-023-03845-1

**Published:** 2023-10-25

**Authors:** Lei Jin, Lingtong Shan, Kaiyan Yu, Yilin Pan, Yangyang Sun, Jiapeng Chen, Lixiang Han, Wei Li, Zhi Li, Yangyang Zhang

**Affiliations:** 1 Department of Critical Care Medicine, Shanghai Chest Hospital, Shanghai Jiao Tong University School of Medicine, Shanghai, China; 2 Department of Thoracic Surgery, Sheyang County People’s Hospital, Yancheng, China; 3 The First Clinical Medical College of Nanjing Medical University, Nanjing, China; 4 Xinglin College, Nantong University, Nantong, China; 5 Department of Cardiovascular Surgery, Jiangsu Province Hospital, the First Affiliated Hospital of Nanjing Medical University, 300 Guangzhou Road, Nanjing, 210029 China; 6 Department of Cardiovascular Surgery, Shanghai Chest Hospital, Shanghai Jiao Tong University School of Medicine, 241 Huaihai Road, Shanghai, 200030 China

**Keywords:** Elderly patients, Acute kidney injury, Coronary artery bypass grafting, Glomerular filtration rate, Risk factor

## Abstract

**Purpose:**

To explore the incidence of postoperative acute kidney injury (AKI) after coronary artery bypass grafting (CABG) in elderly Chinese patients (≥ 75 years old) and its impacts on the short- and long-term prognosis.

**Methods:**

A total of 493 patients aged 75–88 years old who underwent CABG from two medical centers between January 2006 and October 2021 were involved. Perioperative (preoperative and 7 days after operation) serum creatinine (Scr) levels were measured in all the enrolled patients. Univariate and multivariate logistic regression analyses were conducted to explore the independent risk factors of postoperative in-hospital mortality. Kaplan–Meier curves and COX model were used to test the risk factors of all-cause death during follow-up. Propensity score matching was used to balance differences between AKI and control groups. The primary outcome event was in-hospital death, and the secondary outcome was all-cause death during follow-up.

**Results:**

The 198 patients were diagnosed with postoperative AKI. Intra-aortic balloon pump (IABP), cardiopulmonary bypass, and postoperative AKI were independent risk factors of in-hospital death. Gender, New York Heart Association Classification, preoperative eGFR, last eGFR within 7 days after operation, postoperative AKI, and postoperative renal function all impacted long-term prognosis. After 1:1 matching, 190 patients were included in the AKI and control groups. Use of IABP, use of cardiopulmonary bypass, and occurrence of postoperative AKI were still independent risk factors of in-hospital death. Preoperative eGFR, last eGFR within 7 days after operation, postoperative AKI and postoperative renal function all impacted long-term prognosis.

**Conclusion:**

The incidence of postoperative AKI in elderly patients undergoing CABG is high, and postoperative AKI is an independent risk factor of both short- and long-term postoperative prognosis.

## Introduction

With the aging society, the prevalence of coronary atherosclerotic heart diseases remains high in China. Coronary artery bypass grafting (CABG) is one of the important methods for surgical treatment of coronary artery heart diseases complicated with cardiac insufficiency [1]. Patients undergoing CABG tend to be advanced ageing [2]. Compared to young patients, old patients are largely different in pathophysiology, disease characteristics and prognosis [3, 4]. However, due to the high requirements of surgical technology and the complexity of perioperative management, these patients undergoing CABG frequently suffer perioperative serious complications [5]. Among these complications, acute kidney injury (AKI) is a particular concern to cardiac surgeons owing to its high incidence [6, 7]. Postoperative multiple organ dysfunction, the most common of which is cardiac insufficiency combined with AKI, is closely related to in-hospital mortality and postoperative quality of life [8, 9].

Many scholars focus on the occurrence and treatment of postoperative AKI in patients undergoing CABG [10, 11]. Reportedly, the occurrence of postoperative AKI is an independent risk factor of in-hospital mortality in CABG-treated patients [12]. Age is another independent risk factor of AKI development after CABG [13]. Postoperative AKI in elderly CABG-treated patients will be a catastrophe. However, the incidence, risk factors and impact of AKI on the short- and long-term prognosis of elderly Chinese patients undergoing CABG have been rarely reported.

The purpose of this study was to clarify the incidence of AKI after CABG in elderly Chinese patients more than 75 years old and its relationship with the short- and long-term prognosis, and to further clarify the corresponding independent risk factors.

## Materials and methods

### Study design

This was a retrospective cohort and observational study. Patients were selected from two tertiary hospitals in east China between January 2006 and October 2021.

### Patients

**Fig. 1:**
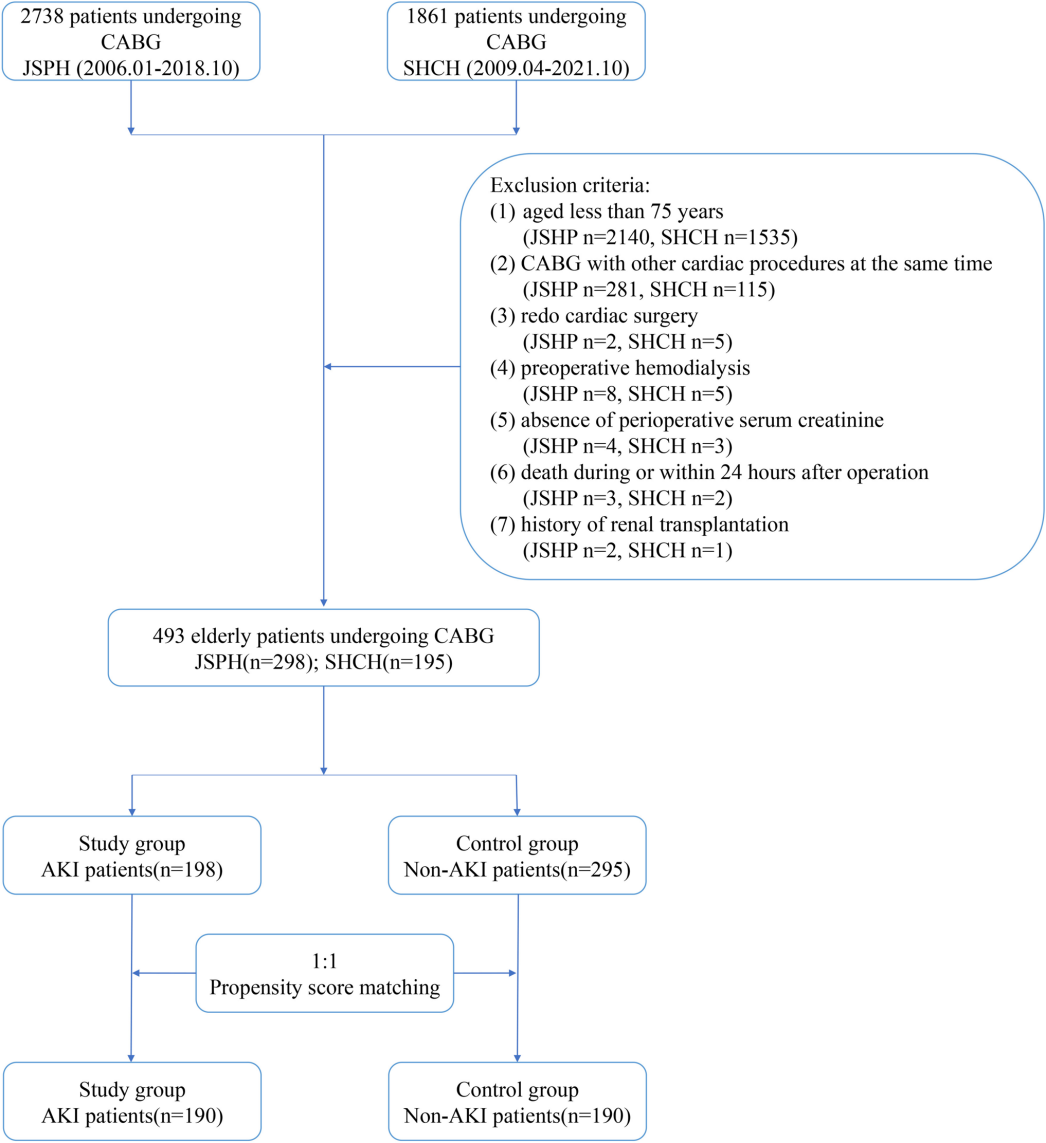
Patients’ enrollment flowchart. *JSPH* Jiangsu Province Hospital, *SHCH* Shanghai Chest Hospital, *CABG* coronary artery bypass grafting, *AKI* acute kidney injury

This study involved 4599 consecutive patients who underwent CABG at one of two medical centers (Jiangsu Province Hospital, and Shanghai Chest Hospital). Exclusion criteria were (1) age less than 75 years; (2) CABG with other cardiac procedures simultaneously; (3) redo cardiac surgery; (4) preoperative hemodialysis; (5) severe absence of medical records, especially serum creatinine (Scr); (6) death during or within 24 h after operation; (7) history of renal transplantation. After a rigorous inclusion and exclusion process, 493 patients made up the original database for this study. The patient selection is shown in Fig. 1.

### Serum creatinine

Venous blood of all patients both before and 7 days after operation was collected to measure Scr levels in the central laboratories of the hospitals. To reduce the effect of the contrast medium on the kidneys, the baseline Scr levels were measured 5 days after coronary angiography and within 48 h before surgery. Patients were divided into two groups depending on whether postoperative AKI occurred: an AKI group and a control group. The baseline, intraoperative and postoperative data of the patients were obtained from hospital information systems, and were entered by two medical students who did not know the grouping information.

### Follow-up

All patients who survived were followed up after the procedure. Follow-up visits were paid in the form of telephone, outpatient and written follow-ups. The member in charge of the follow-up visits guided each patient on postoperative rehabilitation and collected relevant information. In particular, patients who died during the follow-up visits were registered, and the cause of death was recorded. The latest follow-up date was 29 December, 2021.

### Informed consent

All included patients or their legal representatives signed a written informed consent for all surgical procedures. In this retrospective study, there was no risk of disclosure of patient privacy or violation of patient life and health. With the approval and consent of the Ethics Committees, all enrolled patients were exempted from informed consent for their participation. This study was approved by the Ethics Committees of both hospitals and was registered with the number of ChiCTR2200061191 (http://www.chictr.org.cn/).

### Definition of key indicators

#### Postoperative AKI

The clinical practice guideline from Kidney Disease: Improving Global Outcomes (KDIGO) [8] defines postoperative AKI as an increase in Scr level (1) by ≥ 0.3 mg/dl (≥ 26.5 μmol/l) within 48 h, or (2) by ≥ 1.5 times from the baseline, which is known or presumed to occur within the prior 7 days. AKI is staged by the Scr level for severity as follows: Stage 1: 1.5–1.9 times from the baseline or an increase ≥ 0.3 mg/dl (≥ 26.5 umol/l); Stage 2: 2.0–2.9 times from the baseline; Stage 3: 3.0 times from the baseline or an increase ≥ 4.0 mg/dl (≥ 353.6 umol/l) or initiation of renal replacement therapy.

#### In-hospital death

In-hospital death was defined as any death within 30 days after operation or during postoperative hospitalization.

#### Recovery of renal function after surgery

Based on previous studies [14, 15] and clinical practice, recovery of renal function, also called restoration of renal function, after surgery was defined as an estimated glomerular filtration rate (eGFR) larger than or equal to 60 mL/min/1.73m^2^. Recovery of renal function was considered to be achieved when a patient was diagnosed with postoperative AKI and had an eGFR larger than or equal to 60 mL/min/1.73m^2^ detected prior to discharge. The entire cohort was divided into a recovered group and a non-recovered group depending on whether renal function was restored.

#### eGFR

Among many formulas for eGFR, our previous research demonstrates the Cockcroft-Gault (CG) equation is more suitable for Chinese patients undergoing CABG [16]. The CG equation is shown as follows:

$$eGFR=Cr*0.84*1.73/BSA$$

$$Cr=\frac{140-age}{72*Scr\,(mg/dl)}*weight\,\left(kg\right)*\left(0.85\, if\, female\right)$$

$${BSA\,({m}^{2})=weight}^{0.425}*{height\,(cm)}^{0.725}*0.007184$$

where BSA is the body surface area.

#### Valvular disease

Valvular disease is defined as moderate valvular insufficiency that does not require surgical treatment.

### Outcomes

The primary outcome event was in-hospital death, and the secondary outcome was all-cause death during follow-up.

### Statistical analysis

Continuous variables were expressed as median and interquartile ranges (IQR) or mean ± standard deviation, and were analyzed by Student’s t-test in case of normal distribution or by Mann–Whitney U test in case of non-normal distribution. Categorical variables were expressed as number and percentage of patients and analyzed with Chi-square or Fisher exact test, as appropriate.

Univariate and multivariate logistic regression analyses were used to identify independent risk factors for in-hospital death and postoperative AKI using odds ratio (OR) and 95% confidence intervals (CIs). To avoid overfitting in the model, the significant variables identified by univariate analysis were further evaluated in multivariate analysis. Variables with *P* < 0.1 in univariate analysis and the clinically recognized risk factors were included in the multivariate analysis.

Kaplan–Meier curves were used to estimate the event-free survival rates in the two groups. Differences in survival rates between groups were analyzed with log-rank tests. A COX proportional hazards model was built with survival outcome and survival time as the response variables, and it allowed for simultaneous analysis of the impact of numerous factors on survival and can analyze information with truncated survival time.

As this was a retrospective observational study, some of the variables were unevenly distributed between the two groups, which may skew the findings. Hence, propensity score matching was used to adjust for between-group differences to make the two groups comparable. To further exclude the effects of confounding factors, we used one-to-one propensity score matching to adjust and screen pairs for baseline information for both groups. Each patient suffering postoperative AKI was matched to a patient without postoperative AKI using these variables: age, gender, hypertension, preoperative eGFR, intra-aortic balloon pump (IABP) implantation, pulmonary hypertension, previous percutaneous coronary intervention (PCI), coronary artery disease (CAD) classification and EuroSCORE II. The closed propensity score on the logit scale with a caliper was 0.05.

All analyses were two-sided, with the significant level at *P* < 0.05. Data were analyzed with SPSS 25.0 (IBM Corp, Armonk, NY). Figures were plotted on GraphPad Prism 9.0 (GraphPad Software, CA).

## Results

### Baseline clinical characteristics

In the total cohort, the median age was 76 years old. There were 369 (74.85%) males, 354 (71.81%) patients with hypertension, 155 (31.44%) patients with diabetes, 19 (3.85%) patients receiving emergency operation, 57 (11.56%) patients with valvular disease, and 57 (11.56%) patients with history of PCI. Thirty (6.09%) deaths happened within 30 days after operation.

After one-to-one propensity score matching, 190 patients were selected in each of the control and AKI groups. After propensity score matching, some of the factors that differed between groups were corrected and the differences between groups were not significant.

The in-hospital death increased in the AKI group (12.12% vs. 2.03%, *P* < 0.001). The AKI group compared with the control group had older age (77 vs. 76, *P* = 0.033), higher preoperative Scr level (87.00 vs. 81.60, *P* = 0.001), higher proportions of hypertension (76.77% vs. 68.47%, *P* = 0.045), IABP implantation (7.07% vs. 2.03%, *P* = 0.024), and previous PCI (15.15% vs. 9.15%, *P* = 0.041). After propensity score matching, the significant differences in mortality remained between the two groups (11.58% vs 2.63%, *P* < 0.001). Baseline clinical characteristics of the total cohort and the two groups were shown in Table 1.

**Table 1:** Baseline clinical characteristics of total cohort and subsets before and after propensity score matching

	Before matching			After matching		
	Total (n = 493)	AKI group (n = 198)	Control group (n = 295)	Total (n = 380)	AKI group (n = 190)	Control group (n = 190)
Age (y)	76.00 (3.00)	77.00 (4.00)	76.00 (3.00)	77.00 (4.00)	77.00 (4.00)	76.00 (4.00)
Gender (male) (n, %)	369 (74.85)	152 (76.77)	217 (73.56)	272 (71.58)	146 (76.84)	126 (66.32)
Weight (kg)	65.00 (14.50)	65.00 (16.00)	66.00 (14.00)	65.00 (14.00)	65.00 (16.00)	64.00 (13.00)
Height (cm)	166.00 (10.00)	167.50 (10.00)	165.00 (11.00)	165.00 (11.00)	167.50 (10.00)	165.00 (12.00)
BMI (kg/m^2^)	24.03 (4.14)	23.88 (3.97)	24.09 (4.26)	23.86 (4.07)	24.04 (4.00)	23.47 (4.18)
BSA (m^2^)	1.82 (0.23)	1.81 (0.24)	1.82 (0.23)	1.80 (0.24)	1.83 (0.24)	1.78 (0.22)
Morbid obesity (n, %)	25 (5.07)	9 (4.55)	16 (5.42)	16 (4.21)	9 (4.74)	7 (3.68)
NYHA IV (n, %)	26 (5.27)	14 (7.07)	12 (4.07)	22 (5.79)	12 (6.32)	10 (5.26)
CAD classification						
Stable angina (n, %)	148 (30.02)	52 (26.26)	96 (32.54)	113 (29.74)	51 (26.84)	62 (32.63)
Unstable angina (n, %)	286 (58.01)	118 (59.60)	168 (56.95)	216 (56.84)	112 (58.95)	104 (54.74)
AMI (n, %)	59 (11.97)	28 (14.14)	31 (10.51)	51 (13.42)	27 (14.21)	24 (12.63)
Hypertension (n, %)	354 (71.81)	152 (76.77)	202 (68.47)	278 (73.16)	144 (75.79)	134 (70.53)
Diabetes (n, %)	155 (31.44)	67 (33.84)	88 (29.83)	118 (31.05)	65 (34.21)	53 (27.89)
Cerebrovascular disease (n, %)	116 (23.53)	42 (21.21)	74 (25.08)	87 (22.89)	42 (22.11)	45 (23.68)
Preoperative Scr (μmol/l)	83.80 (29.00)	87.00 (38.00)	81.60 (24.50)	81.15 (30.30)	86.90 (37.65)	76.00 (27.35)
Preoperative eGFR (mL/min/1.73 m^2^)	59.98 (22.08)	56.10 (22.37)	62.56 (22.15)	59.72 (22.18)	57.27 (21.11)	62.27 (22.59)
Preoperative LVEF (%)	61.90 (8.00)	61.00 (8.72)	62.40 (7.20)	61.65 (7.80)	61.00 (8.65)	61.90 (7.00)
Number of diseased vessels (n, %)						
1	20 (4.06)	7 (3.54)	13 (4.41)	15 (3.95)	6 (3.16)	9 (4.74)
2	56 (11.36)	26 (13.13)	30 (10.17)	46 (12.11)	24 (12.63)	22 (11.58)
3	417 (84.58)	165 (83.33)	252 (85.42)	319 (83.95)	160 (84.21)	159 (83.68)
Peripheral vascular disease (n, %)	44 (8.92)	22 (11.11)	22 (7.46)	33 (8.68)	21 (11.05)	12 (6.32)
Surgical status (n, %)						
Emergency	19 (3.85)	6 (3.03)	13 (4.41)	14 (3.68)	6 (3.16)	8 (4.21)
Rescue	2 (0.41)	2 (1.01)	0 (0.00)	1 (0.26)	1 (0.53)	0 (0.00)
Valvular disease (n, %)	57 (11.56)	22 (11.11)	35 (11.86)	43 (11.32)	21 (11.05)	22 (11.58)
IABP implantation (n, %)	20 (4.06)	14 (7.07)	6 (2.03)	13 (3.42)	10 (5.26)	3 (1.58)
COPD (n, %)	46 (9.33)	23 (11.62)	23 (7.80)	37 (9.74)	22 (11.58)	15 (7.89)
Atrial fibrillation (n, %)	21 (4.26)	12 (6.06)	9 (3.05)	16 (4.21)	11 (5.79)	5 (2.63)
Pulmonary hypertension (n, %)	176 (35.70)	83 (41.92)	93 (31.53)	145 (38.16)	78 (41.05)	67 (35.26)
Previous PCI (n, %)	57 (11.56)	30 (15.15)	27 (9.15)	44 (11.58)	24 (12.63)	20 (10.53)
Bypass graft number (n)	3.00 (1.00)	3.00 (1.00)	3.00 (1.00)	3.00 (1.00)	3.00 (1.00)	3.00 (1.00)
Cardiopulmonary bypass (n, %)	42 (8.52)	21 (10.61)	21 (7.12)	35 (9.21)	20 (10.53)	15 (7.89)
In-hospital mortality (n, %)	30 (6.09)	24 (12.12)	6 (2.03)	27 (7.11)	22 (11.58)	5 (2.63)
EuroSCORE II	2.42 (1.93)	2.68 (2.25)	2.34 (1.69)	2.46 (2.09)	2.51 (2.15)	2.43 (1.93)

*AKI* Acute kidney injury, *BMI* body mass index, *BSA* body surface area, *NYHA* New York heart association, *CAD* coronary artery disease, *AMI* acute myocardial infarction, *Scr* Serum creatinine, eGFR estimated glomerular filtration rate, *LVEF* left ventricular ejection fraction, *IABP* intra-aortic balloon pump, *COPD* chronic obstructive pulmonary disease, *PCI* percutaneous coronary intervention, *EuroSCORE II* European system for cardiac operative risk evaluation II

About 198 of the 493 patients (40.16%) were diagnosed with postoperative AKI according to the KDIGO guideline, including 149, 30 and 19 patients at stage 1, 2 and 3 respectively. Among the 198 patients, stage 1 and severe stage 3 accounted for the majority (75.25%) and 9.60% respectively.

### Trends in Scr

**Fig. 2:**
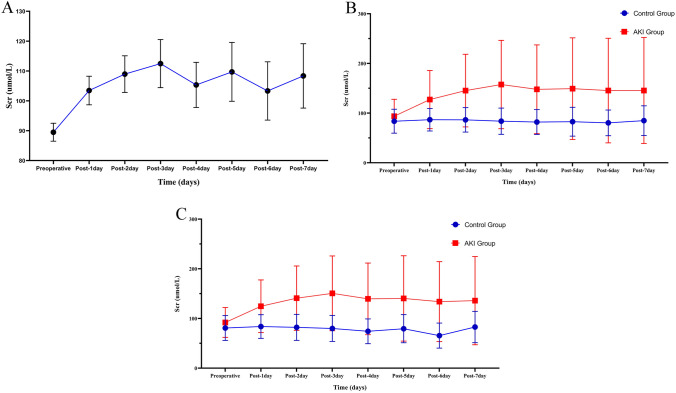
The median values of serum creatinine in **A** overall patients, **B** AKI group and control group before PSM and **C** AKI group and control group after PSM. *AKI* acute kidney injury, *PSM* propensity score matching

In the total cohort, postoperative Scr levels generally increased from the preoperative levels. Also, at the 7 time points after operation, Scr levels increased, then decreased and finally leveled off. The highest peak in Scr level occurred on the third postoperative day (Fig. 2A).

In the two groups, Scr levels changed in similar trends. However, the Scr levels in the AKI group fluctuated more and were higher than the control group at any time point (Fig. 2B). Even after propensity score matching, the Scr levels maintained the same trends (Fig. 2C).

**Fig. 3:**
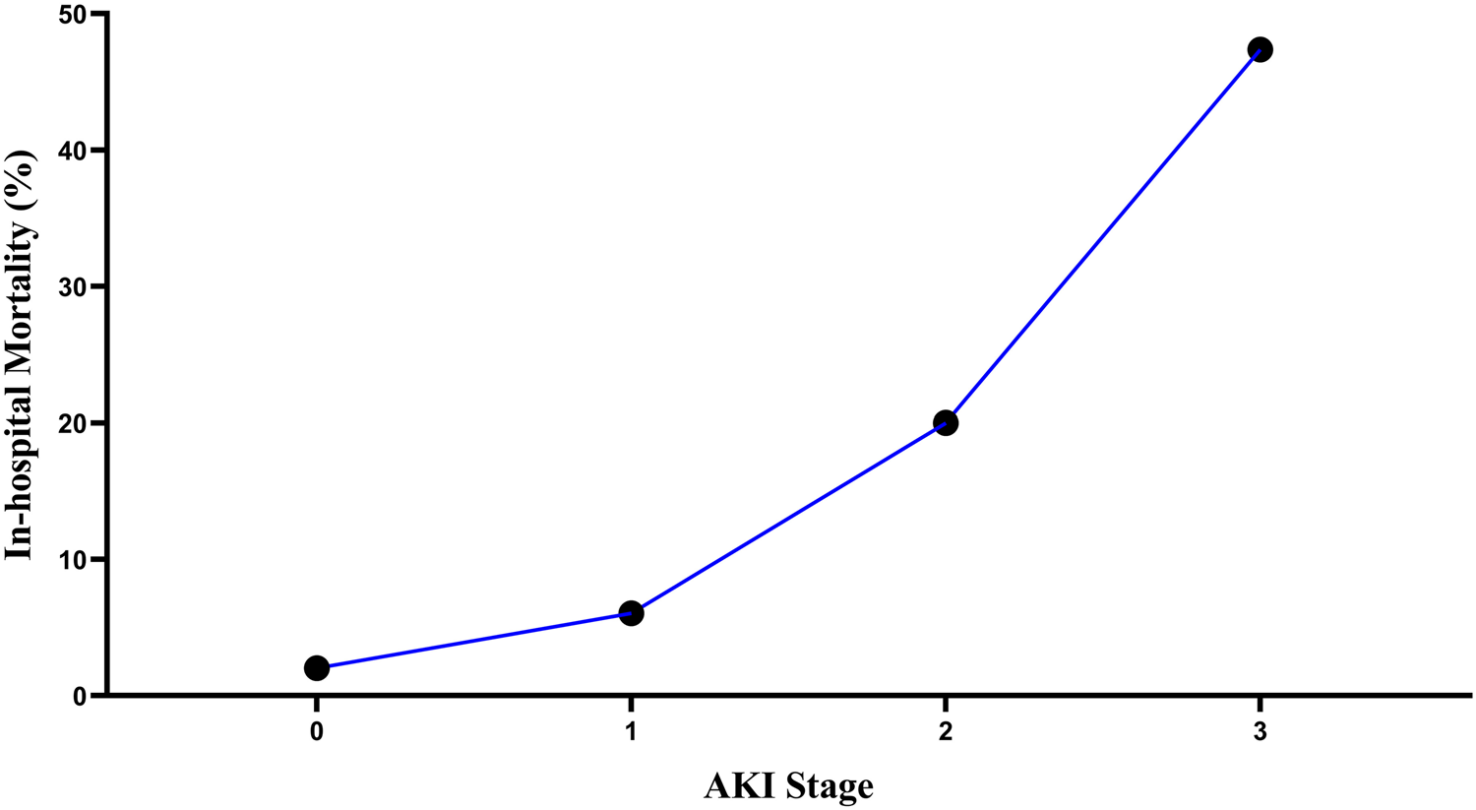
In-hospital mortality of patients undergoing CABG in different AKI stages

Figure 3 shows the in-hospital mortality of patients undergoing CABG in different AKI stages. As the AKI stage proceeds, the in-hospital mortality rate of patients increases sharply.

### Impact of risk factors on in-hospital death

Univariate logistic analysis showed that New York Heart Association (NYHA) classification, preoperative eGFR, number of diseased vessels, emergency surgery, IABP implantation, cardiopulmonary bypass, and postoperative AKI were risk factors of in-hospital death (Table 2). Furthermore, multivariate logistic regression analysis showed that IABP implantation, cardiopulmonary bypass, and postoperative AKI were independent risk factors of in-hospital death (Table 2). After propensity score matching, IABP implantation, renal failure, cardiopulmonary bypass, and postoperative AKI were the independent risk factors of in-hospital death (Table 3).

**Table 2:** Risk factors of In-hospital death by univariate and multivariate logistic regression analysis

	Univariate analysis			Multivariate analysis		
	OR	95% CI	P	OR	95% CI	P
Age	1.124	0.991–1.275	0.069	1.025	0.871–1.206	0.767
Gender	1.297	0.578–2.912	0.529	0.874	0.314–2.431	0.796
Morbid obesity	0.882	0.649–1.119	0.422			
NYHA classification	2.256	1.299–3.918	0.004	1.741	0.857–3.467	0.114
CAD classification	1.138	0.630–2.055	0.668			
Hypertension	0.772	0.352–1.695	0.52			
Diabetes	1.998	0.949–4.205	0.068	1.893	0.768–4.667	0.166
Cerebrovascular disease	0.669	0.320–1.400	0.286			
Preoperative eGFR	0.962	0.940–0.985	0.001	0.981	0.954–1.008	0.161
Preoperative LVEF	0.965	0.928–1.002	0.066	1.009	0.995–1.065	0.755
Number of diseased vessels	0.517	0.295–0.909	0.022	0.48	0.178–1.297	0.148
Peripheral vascular disease	1.627	0.541–4.895	0.386			
Surgical status	3.603	1.382–9.396	0.009	2.838	0.804–10.018	0.105
Valvular disease	1.19	0.400–3.543	0.754			
IABP implantation	3.18	2.010–5.032	< 0.001	2.113	1.224–3.646	0.007
COPD	0.68	0.157–2.952	0.607			
Atrial fibrillation	2.747	0.762–9.904	0.123			
Pulmonary hypertension	1.407	0.871–2.273	0.163			
Previous PCI	2.02	0.788–5.174	0.143			
Bypass graft number	0.719	0.489–1.058	0.095	1.083	0.590–1.990	0.796
Cardiopulmonary bypass	4.588	1.901–11.076	0.001	3.595	1.158–11.160	0.027
Medical Center	1.254	0.866–1.815	0.231			
Postoperative AKI	3.556	2.421–5.221	< 0.001	2.94	1.883–4.590	< 0.001

*OR* odds ratio, *CI* confidence interval, *NYHA* New York heart association, *CAD* coronary artery disease, *eGFR* estimated glomerular filtration rate, *LVEF* left ventricular ejection fraction, *IABP* intra-aortic balloon pump, *COPD* chronic obstructive pulmonary disease, *PCI* percutaneous coronary intervention, *AKI* acute kidney injury

**Table 3:** Risk factors of In-hospital death by univariate and multivariate logistic regression analysis after propensity score matching

	Univariate analysis			Multivariate analysis		
	OR	95% CI	*P*	OR	95%CI	*P*
Age	1.102	0.962–1.262	0.16	1.098	0.932–1.293	0.265
Gender	1.065	0.452–2.512	0.885	1.034	0.389–2.750	0.947
BMI	0.980	0.863–1.113	0.76			
BSA	0.567	0.052–6.158	0.641			
Morbid obesity	0.945	0.685–1.304	0.732			
NYHA classification	1.855	1.036–3.319	0.038	1.809	0.901–3.634	0.096
CAD classification	1.041	0.563- 1.923	0.899			
Hypertension	0.599	0.265–1.356	0.219			
Diabetes	2.193	0.997–4.826	0.051	1.937	0.777–4.825	0.156
Cerebrovascular disease	0.531	0.214–1.314	0.171			
Preoperative eGFR	0.906	0.878–0.935	< 0.001			
Preoperative LVEF	0.978	0.934–1.023	0.333			
Number of diseased vessels	0.621	0.326–1.186	0.149			
Peripheral vascular disease	1.943	0.629–6.001	0.248			
Surgical status	2.748	0.816–9.527	0.103			
Valvular disease	0.978	0.282–3.395	0.972			
IABP implantation	3.594	2.000–6.459	< 0.001	3.309	1.668–6.563	0.001
COPD	0.339	0.045–2.571	0.295			
Atrial fibrillation	1.937	0.417–9.002	0.399			
Pulmonary hypertension	0.919	0.504–1.675	0.782			
Previous PCI	1.361	0.448–4.136	0.587			
Renal failure	7.066	2.256–22.131	0.001	5.272	1.337–20.793	0.018
Bypass graft number	0.770	0.510–1.164	0.215			
Cardiopulmonary bypass	4.062	1.582–10.435	0.004	3.23	1.055–9.893	0.04
Medical Center	1.084	0.731–1.608	0.689			
EuroSCORE II	1.135	1.029–1.252	0.011	0.965	0.837–1.112	0.622
Postoperative AKI	4.845	1.795–13.082	0.002	3.993	1.339–11.906	0.013

*OR* odds ratio, *CI* confidence interval, *BMI* body mass index, *BSA* body surface area, *NYHA* New York heart association, CAD, coronary artery disease, *eGFR* estimated glomerular filtration rate, *LVEF* left ventricular ejection fraction, *IABP* intra-aortic balloon pump, *COPD* chronic obstructive pulmonary disease, *PCI* percutaneous coronary intervention, *EuroSCORE II* European system for cardiac operative risk evaluation II, *AKI* acute kidney injury

### Impact of risk factors on long-term survival

The medium duration of follow-up was 56 months (interquartile range: 25–80 months). The Kaplan–Meier curves showed the 1-, 3- and 5-year survival rates (non-AKI vs. AKI) were 94.5% (96.4% vs. 91.1%), 90.1% (93.1% vs. 86.7%), and 84.3% (88.6% vs. 82.3%) respectively.

**Fig. 4:**
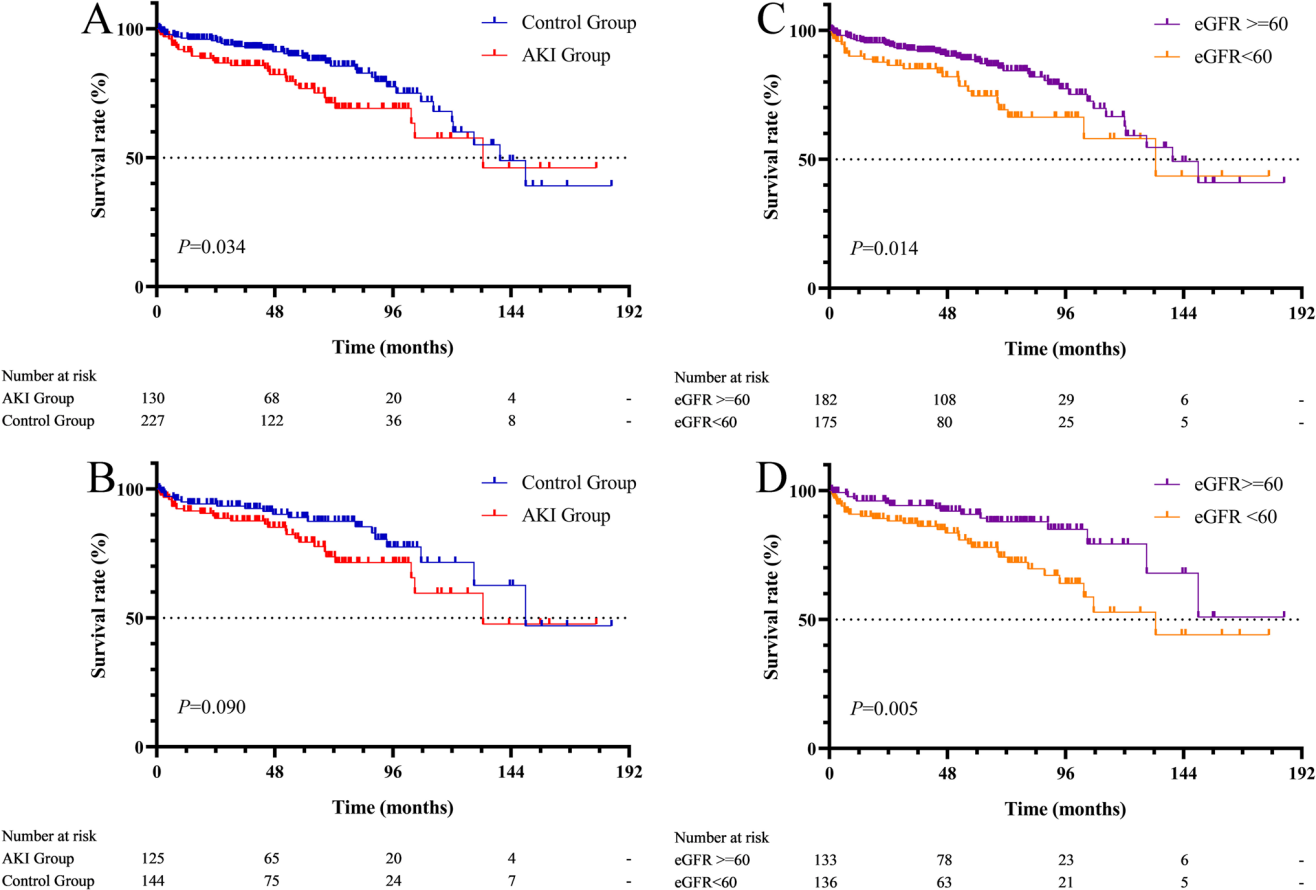
Impact of AKI and postoperative renal function on long-term survival. **A** Overall Kaplan–Meier survival curve between the AKI group and control group before PSM; **B** Overall Kaplan–Meier survival curve between the AKI group and control group after PSM; **C** Overall Kaplan–Meier survival curve between whether or not postoperative renal function is restored before PSM; **D** Overall Kaplan–Meier survival curve between whether or not postoperative renal function is restored after PSM. *AKI* acute kidney injury, *PSM* propensity score matching, *eGFR* estimated glomerular filtration rate

The Kaplan–Meier curves uncovered a worse long-term prognosis in the AKI group relative to the control group (Fig. 4A) and in the renal function non-recovery group relative to the recovery group. The differences were significant (Fig. 4C) and existed after propensity score matching (Fig. 4B, D).

**Fig. 5:**
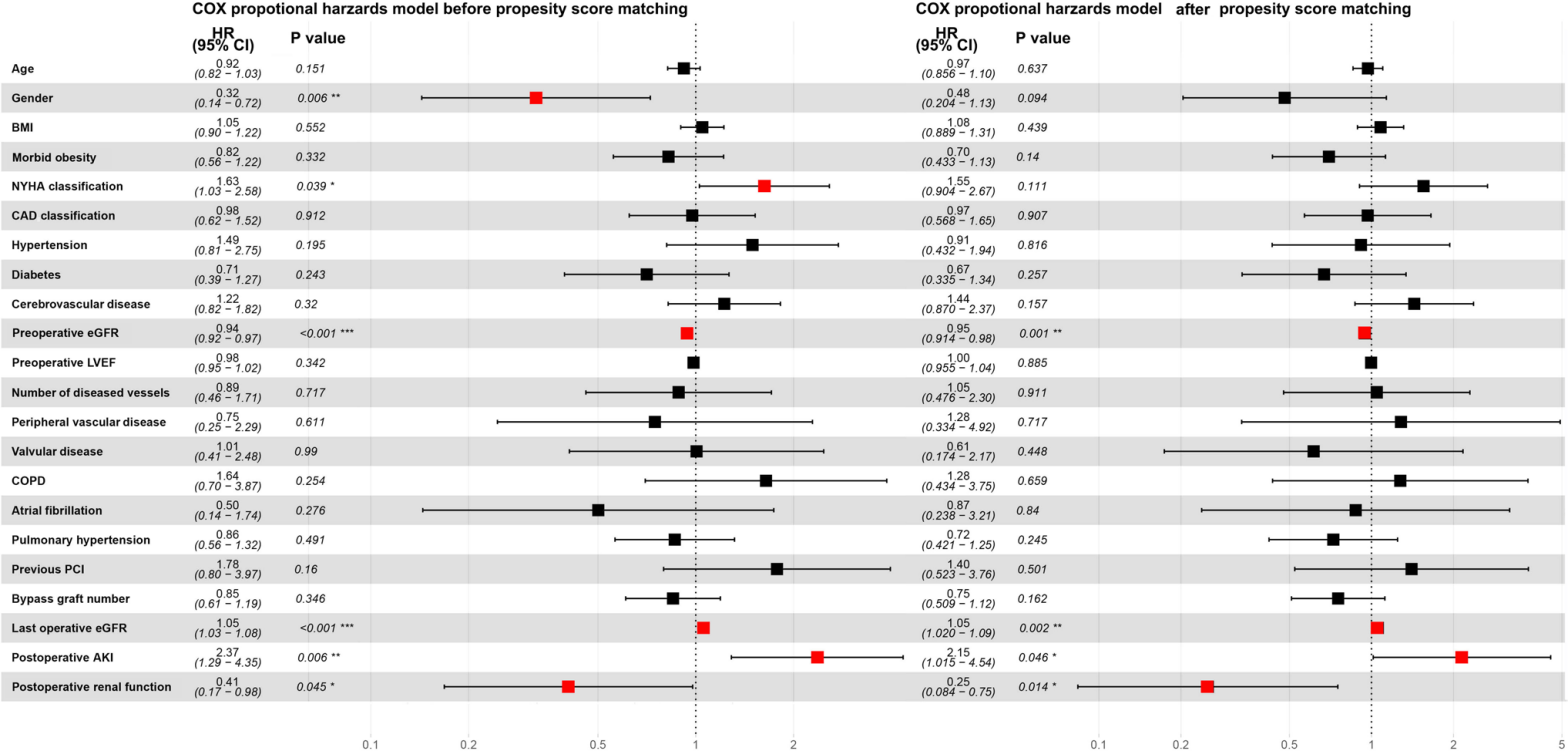
Forest plots of independent risk factors of long-term mortality before and after propensity score matching

The COX proportional model suggests that gender, NYHA classification, preoperative eGFR, last eGFR within 7 days after operation, postoperative AKI and postoperative renal function all impact long-term prognosis. After propensity score matching, preoperative eGFR, the last eGFR within 7 days after operation, postoperative AKI and postoperative renal function all impact long-term prognosis (Fig. 5).

## Discussion

This clinical observational study demonstrates the incidence of postoperative AKI is very high (40.16%) in the elderly patients (over 75 years old). Although most (75.15%) of the postoperative AKI patients are at stage 1, postoperative AKI is an independent risk factor of in-hospital death and long-term mortality in the elderly patients.

In recent years, an increasing number of CAD patients have undergone CABG [17]. CABG is an effective treatment for CAD and has greatly improved the quality of life and prognosis of patients. Atherosclerosis, a disease of the elderly, is featured by increasing comorbidities and decreasing reserve function of all organs of the body, and significantly reduces body tolerance to surgery [18]. With the global social aging, China will inevitably face more and more elderly CAD patients with heart surgery who are particularly older than these with valvular and aortic diseases. They are likely to suffer postoperative complications, such as low cardiac output syndrome, respiratory infection, stroke and renal insufficiency [19].

Postoperative AKI is one of the most common and serious complications after CABG [20]. A registry study about CABG in China and a survey in India show that AKI occurs in 12%-48% of patients undergoing CABG, which in turn leads to increased mortality and reduced quality of life, especially for patients with combined heart failure [15, 21, 22]. However, little literature has reported the characteristics of renal function changes after CABG and the corresponding impacts on the short- and long- term prognosis of elderly patients [23, 24].

In this project involving 493 cases, the incidence of postoperative AKI is high, which is because the included patients are all elderly. Moreover, the AKI group has older age, higher preoperative Scr level, higher in-hospital mortality, and higher proportions of hypertension, IABP implantation, and previous PCI. Similar results can be found in other studies on elderly cardiac surgery [25–27]. All of these factors may influence the renal function changes in patients after cardiac surgery.

The data of this study confirm postoperative AKI as an independent risk factor of in-hospital mortality in elderly patients. The occurrence of postoperative AKI is indicative of poor prognosis, which will inform clinical practice of elderly patients. After adjustment for other factors, postoperative AKI remains an independent risk factor in elderly patients. Another study shows that postoperative AKI impacts long-term prognosis [24]. Aggressive postoperative correction of renal function may also have a beneficial effect on patient prognosis. The reason may be that some of the postoperative AKI patients have uncorrected renal function, especially for elderly patients, and develop chronic renal failure, which more greatly impact the overall organ function of patients.

The existing diagnosis of acute renal failure (ARF) lacks widely-accepted criteria and ignores the pathophysiological changes that occur in the early stages of kidney damage [28]. As a result, AKI is gradually used to replace ARF, expect that it will be identified at an early stage of kidney injury and that a uniform diagnostic staging criterion will be given. The most commonly-used diagnostic criteria are RIFLE [29], AKIN [30] and KDIGO [31]. KDIGO combines the advantages of RIFLE and AKIN and is widely used in clinical practice [32]. In this study, KDIGO was used to diagnose AKI in patients undergoing CABG. According to the diagnostic criteria, AKI is a relative indicator of the changes of renal function. AKI is both connected with and different from eGFR, an absolute indicator of renal function. Preoperative low eGFR was found as an independent risk factor of postoperative AKI. Among the surviving patients with AKI, the recovery rate within 7 days after operation was only 26.92% (35/130), which is because the recovery of renal function in elderly AKI patients is a long process [33, 34]. This study only observed the Scr level within 7 days after operation. More AKI patients shall recover renal function in the follow-up time. Interestingly, compared with postoperative AKI, postoperative renal function is also independent risk factor of long-term prognosis, regardless of propensity score matching. However, recovery of renal function is an absolute indicator of postoperative eGFR more than 60 mL/min/1.73m^2^ in elderly patients. In clinical work, it is important not to look at a single indicator, but to analyze in the context of the patient's specific situation.

In this study, less than 10% of patients underwent CABG with cardiopulmonary bypass. This approach is based on the preference of surgeons and medical centers. Most operations of CABG in China rely on the off-pump surgical technique [35–37]. Admittedly, if surgeons consider a patient’s condition is serious and needs more revascularization, they often choose the on-pump operation. In other words, patients with severe conditions are more likely to undergo cardiopulmonary bypass. This preference may result in the relatively high in-hospital death of the on-pump CABG patients.

Reportedly, cardiopulmonary bypass has no significant effect on in-hospital death, but it is a risk factor of postoperative AKI [38]. This finding is different from our study, because of some differences between the two studies. In the other study, the average age of the patients is 62 years old and the ratio of the on-pump technique is high as 74.9% [38]. In our study, however, all patients are aged 75 years and above, and the ratio of the on-pump route is only 8.5%. These reasons may lead to the differences above.

There are also some limitations. Firstly, in this retrospective observational study, it is difficult to control for bias and for us to apply the propensity score matching. Secondly, for patients in the general ward with normal creatinine levels after CABG, daily measurement was impractical and uneconomical, so not all patients had their serum creatinine measured 7 times after surgery. Thirdly, patients came from two medical centers and the inclusion was over a long period, so differences in medical and postoperative care may be confounding factors.

## Conclusions

The incidence of postoperative AKI in elderly patients undergoing CABG is quite high, which is associated with reduced short- and long-term survival.

## Data Availability

The original contributions presented in the study are included in the article/supplementary material, further inquiries can be directed to the corresponding authors.

## References

[1] Wintgen L, Dakkak AR, Shakaki MA (2021). Acute kidney injury following coronary artery bypass grafting and control angiography: a comprehensive analysis of 221 patients. Heart Vessels.

[2] Shaefi S, Mittel A, Loberman D, Ramakrishna H (2019). Off-Pump versus on-pump coronary artery bypass grafting-a systematic review and analysis of clinical outcomes. J Cardiothorac Vasc Anesth.

[3] Spadaccio C, Benedetto U (2018). Coronary artery bypass grafting (CABG) vs. percutaneous coronary intervention (PCI) in the treatment of multivessel coronary disease: quo vadis?—a review of the evidences on coronary artery disease. Ann Cardiothorac Surg..

[4] Jovin DG, Katlaps GJ, Sumption KF (2020). Coronary artery bypass graft markers: history, usage, and effects. Gen Thorac Cardiovasc Surg.

[5] Aboul-Hassan SS, Marczak J, Stankowski T (2020). Association between preoperative aspirin and acute kidney injury following coronary artery bypass grafting. J Thorac Cardiovasc Surg.

[6] Sato R, Luthe SK, Nasu M (2017). Blood pressure and acute kidney injury. Crit Care.

[7] Acute FA, Injury K (2018). Nurs Clin North Am.

[8] Ostermann M, Bellomo R, Burdmann EA (2020). Controversies in acute kidney injury: conclusions from a kidney disease: improving global outcomes (KDIGO) conference. Kidney Int.

[9] Lee SA, Cozzi M, Bush EL, Rabb H (2018). Distant organ dysfunction in acute kidney injury: a review. Am J Kidney Dis.

[10] Parlar H, Arıkan AA, Önmez A (2021). Dynamic changes in perioperative cellular inflammation and acute kidney injury after coronary artery bypass grafting. Braz J Cardiovasc Surg.

[11] Li Y, Hou XJ, Liu TS (2021). Risk factors for acute kidney injury following coronary artery bypass graft surgery in a Chinese population and development of a prediction model. J Geriatr Cardiol.

[12] Wang R, Wang X, Zhu Y, Chen W, Li L, Chen X (2020). Acute kidney injury following on-pump or off-pump coronary artery bypass grafting in elderly patients: a retrospective propensity score matching analysis. J Cardiothorac Surg.

[13] Oh TK, Song IA (2021). Postoperative acute kidney injury requiring continuous renal replacement therapy and outcomes after coronary artery bypass grafting: a nationwide cohort study. J Cardiothorac Surg.

[14] Macedo E, Zanetta DM, Abdulkader RC (2012). Long-term follow-up of patients after acute kidney injury: patterns of renal functional recovery. PLoS ONE.

[15] Liu W, Xi Z, Gu C, Dong R, AlHelal J, Yan Z (2018). Impact of major bleeding on the risk of acute kidney injury in patients undergoing off-pump coronary artery bypass grafting. J Thorac Dis.

[16] Li Z, Ge W, Han C (2020). Prognostic values of three equations in estimating glomerular filtration rates of patients undergoing off-pump coronary artery bypass grafting. Ther Clin Risk Manag.

[17] Melly L, Torregrossa G, Lee T, Jansens JL, Puskas JD (2018). Fifty years of coronary artery bypass grafting. J Thorac Dis.

[18] Yue Z, Yan-Meng G, Ji-Zhuang L (2019). Prediction model for acute kidney injury after coronary artery bypass grafting: a retrospective study. Int Urol Nephrol.

[19] Møller CH, Steinbrüchel DA (2014). Off-pump versus on-pump coronary artery bypass grafting. Curr Cardiol Rep.

[20] Gaut JP, Liapis H (2021). Acute kidney injury pathology and pathophysiology: a retrospective review. Clin Kidney J.

[21] Kertai MD, Zhou S, Karhausen JA (2016). Platelet counts, acute kidney injury, and mortality after coronary artery bypass grafting surgery. Anesthesiology.

[22] Nigwekar SU, Kandula P, Hix JK, Thakar CV (2009). Off-pump coronary artery bypass surgery and acute kidney injury: a meta-analysis of randomized and observational studies. Am J Kidney Dis.

[23] Gelsomino S, Del Pace S, Parise O (2017). Impact of renal function impairment assessed by CKD(EPI) estimated glomerular filtration rate on early and late outcomes after coronary artery bypass grafting. Int J Cardiol.

[24] Hillis GS, Croal BL, Buchan KG (2006). Renal function and outcome from coronary artery bypass grafting: impact on mortality after a 2.3-year follow-up. Circulation.

[25] Silveira Santos C, Romani RF, Benvenutti R, Ribas Zahdi JO, Riella MC, Nascimento Do Mazza M (2018). Acute kidney injury in elderly population: a prospective observational study. Nephron.

[26] Lau D, Pannu N, James MT (2021). Costs and consequences of acute kidney injury after cardiac surgery: a cohort study. J Thorac Cardiovasc Surg.

[27] Carrascal Y, Laguna G, Blanco M, Pañeda L, Segura B (2021). Acute kidney injury after heart valve surgery in elderly patients: any risk factors to modify. Braz J Cardiovasc Surg.

[28] Koza Y (2016). Acute kidney injury: current concepts and new insights. J Inj Violence Res.

[29] De Santo LS, Romano G, Galdieri N (2010). RIFLE criteria for acute kidney injury in valvular surgery. J Heart Valve Dis.

[30] Mehta RL, Kellum JA, Shah SV (2007). Acute kidney injury network: report of an initiative to improve outcomes in acute kidney injury. Crit Care.

[31] Stevens PE, Levin A (2013). Evaluation and management of chronic kidney disease: synopsis of the kidney disease: improving global outcomes 2012 clinical practice guideline. Ann Intern Med.

[32] Er RE, Ulusal Okyay G, Aygencel B, Kmaz G, Türko LuM, Erten Y (2020). Comparison between RIFLE, AKIN, and KDIGO: acute kidney injury definition criteria for prediction of in-hospital mortality in critically Ill patients. Iran J Kidney Dis.

[33] Del Giudice A, Aucella F (2012). Acute renal failure in the elderly: epidemiology and clinical features. J Nephrol.

[34] Xu X, Nie S, Liu Z (2015). Epidemiology and clinical correlates of AKI in Chinese hospitalized adults. Clin J Am Soc Nephrol.

[35] Gu Y, Shan L, Liu B (2021). Release profile of cardiac Troponin T and risk factors of postoperative myocardial injury in patients undergoing CABG. Int J Gen Med.

[36] Li X, Shan L, Lv M (2020). Predictive ability of EuroSCORE II integrating cardiactroponin T in patients undergoing OPCABG. BMC Cardiovasc Disord.

[37] Gao F, Shan L, Wang C (2021). Predictive ability of european heart surgery risk assessment system II (EuroSCORE II) and the society of thoracic surgeons (STS) score for in-hospital and medium-term mortality of patients undergoing coronary artery bypass grafting. Int J Gen Med.

[38] Li Z, Fan G, Zheng X (2019). Risk factors and clinical significance of acute kidney injury after on-pump or off-pump coronary artery bypass grafting: a propensity score-matched study. Interact Cardiovasc Thorac Surg.

